# Multiplexing of ChIP-Seq Samples in an Optimized Experimental Condition Has Minimal Impact on Peak Detection

**DOI:** 10.1371/journal.pone.0129350

**Published:** 2015-06-11

**Authors:** Thadeous J. Kacmarczyk, Caitlin Bourque, Xihui Zhang, Yanwen Jiang, Yariv Houvras, Alicia Alonso, Doron Betel

**Affiliations:** 1 Department of Medicine, Division of Hematology/Oncology, Epigenomics Core Facility, Weill Cornell Medical College, New York, New York, United States of America; 2 Department of Surgery, Weill Cornell Medical College, New York, New York, United States of America; 3 Department of Medicine, Weill Cornell Medical College, New York, New York, United States of America; 4 Institute for Computational Biomedicine, Weill Cornell Medical College, New York, New York, United States of America; Northwestern University, UNITED STATES

## Abstract

Multiplexing samples in sequencing experiments is a common approach to maximize information yield while minimizing cost. In most cases the number of samples that are multiplexed is determined by financial consideration or experimental convenience, with limited understanding on the effects on the experimental results. Here we set to examine the impact of multiplexing ChIP-seq experiments on the ability to identify a specific epigenetic modification. We performed peak detection analyses to determine the effects of multiplexing. These include false discovery rates, size, position and statistical significance of peak detection, and changes in gene annotation. We found that, for histone marker H3K4me3, one can multiplex up to 8 samples (7 IP + 1 input) at ~21 million single-end reads each and still detect over 90% of all peaks found when using a full lane for sample (~181 million reads). Furthermore, there are no variations introduced by indexing or lane batch effects and importantly there is no significant reduction in the number of genes with neighboring H3K4me3 peaks. We conclude that, for a well characterized antibody and, therefore, model IP condition, multiplexing 8 samples per lane is sufficient to capture most of the biological signal.

## Introduction

Knowledge of protein-DNA interactions contributes to the understanding of gene expression regulation, and consequently, understanding of biological processes and disease states. The technique of chromatin immunoprecipitation followed by massively parallel sequencing (ChIP-seq) is commonly used for genome-wide identification of protein-DNA interaction sites (e.g. transcription factors), and epigenetic modifications (e.g. histone and DNA modifications) [1, 2]. In contrast to its micro-array predecessor ChIP-ChIP, the ChIP-seq assay provides unbiased genome-wide survey of all protein-DNA interactions and higher genomic resolution of binding site positions. In recent years ChIP-seq has become the primary method for surveying protein-DNA interactions however, it remains a challenging technique to master in part because of vast differences in efficiency of DNA capture. Despite increasing experience and knowledge about the technique [3] there has been no systematic detailed analysis of the impact of sequencing depth on the results of ChIP-seq experiments.

Currently, a single lane on an Illumina HiSeq2500 using v3 chemistry, can typically produce upwards of 150 million (M) single-end reads, often exceeding the sequencing depth needed for many experiments. Combining multiple samples into a single lane, multiplexing, is an economical and efficient way to maximize information content and control cost. The main challenge in designing these experiments is to optimize the multiplexing such that sample coverage is below the saturation point where additional reads do not provide additional information and yet coverage is sufficient to capture the meaningful biological signal. These considerations are highly dependent on the number of binding sites, the quality of the IP procedure and the amount of captured DNA. To improve the accuracy of detecting true IP events most ChIP-seq experiments also sequence the total DNA before IP enrichment, so called “input” sample, in order to control for non-specific chromatin that is purified in the IP.

**Fig 1:**
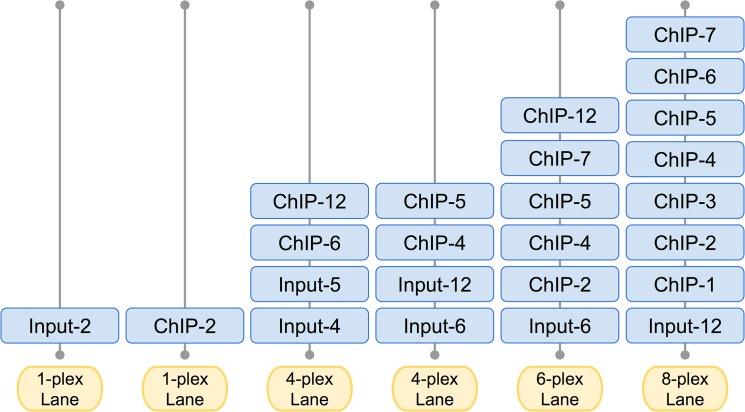
ChIP-seq multiplexing sequencing scheme. The ChIP-seq multiplexing titration scheme consists of: one whole lane of ChIP sample (1-plex), one whole lane of input sample (1-plex), two lanes with 4 samples (4-plex) of 2 ChIP and 2 input samples in each lane, one lane with 6 samples (6-plex) of 1 input and 5 ChIP samples, and one lane with 8 samples (8-plex) of 1 input and 7 ChIP samples. Sample labels correspond to sample type and llumina TruSeq indexed used (e.g. ChIP-5 is IP library with index number 5)

In this study we aimed to characterize the effect of multiplexing ChIP and input samples on the accuracy and number of binding events identified. We selected histone 3-lysine 4 tri-methylation (H3K4me3) as a model histone modification since antibodies that recognize this modification are commercially available and perform well across multiple protocols and labs. ChIP was performed in OCI-LY7 diffuse B-cell lymphoma cells. We evaluated several experimental and computational parameters. Experimentally, to test the effects of barcode indexing, we devised a multiplexing titration scheme starting from one full lane (1-plex) of ChIP and one lane of input samples up to 7 IP samples and one input (8-plex) in a single lane (Fig 1). The remainder of the flow cell contained two lanes of two ChIPs and two inputs (4-plex), one lane of 5 ChIPs and one input (6-plex). This allows for various comparisons between multiplexed and non-multiplexed ChIP as well as different combinations of ChIP and input. Computationally, we evaluated: i) the overlap of the detected peaks with the full lane sequencing, ii) false discovery rates, iii) p-value distributions of detected peaks, iv) genomic coordinates and apex positions of the identified peaks, and v) changes in genomic annotation. We found that when using a highly specific antibody, one can reduce the sequencing coverage down to ~21M reads and retain over 90% of the peaks identified in the non-multiplexed sample (~181M reads) with very little variability or loss of annotated genes.

## Results

To systematically test the effects of multiplexing libraries on the information yield, we performed one preparative ChIP using the highly optimized and specific antibody to trimethylated lysine 4 on histone H3 (H3K4me3)—this histone mark is enriched in the transcriptional start sites of genes– 13 independent libraries were made, 5 from input and 8 from the immunoprecipitated material, and sequenced according to the titration scheme described in Fig 1. Following read mapping and peak identification, there are several comparisons that can be made using this scheme. Throughout the comparisons, the libraries sequenced as 1-plex (Fig 1, 1-plex lane) are used as the reference set. Experiments are labeled as either chip or input with their index, so ChIP with 6^th^ index (from Illumina TruSeq kit) is called chip-6 and input with 4^th^ index is called input-4. Peaks identified by contrasting chip-6 with input-4 are labeled as chip-6::input-4. First, we can compare moderately multiplexed ChIP experiments to data generated from whole lane ChIP and whole lane of input. This allows examining the effect of multiplexing and barcode indexing on peak identification. For example, we can compare peaks identified from ~43M reads (4-plex) of chip-4::input12 with peaks identified from ~21M reads (8-plex) of chip-4::input12 or chip-5::input12. Second, we can compare peaks derived from different combinations of ChIP and input samples from both inter- and intra-lane (see S1 Table for all ChIP/input combinations). For example, there are two 4-plex lanes with 2 ChIPs and 2 inputs, we can compare peaks identified for different combinations of ChIP and input samples such as chip-6::input-4, or chip-6::input-5, chip-6::input-6 and chip-6::input-12. Furthermore, we can test the effect of different amounts of input on peak identification. We can compare peaks identified from ~43M reads of ChIP and either the same number of reads of input or double the number of reads from combined input. As an example, chip-6::input-4 (single input), with chip-6::input-4+input-5 (combined inputs).

**Fig 2:**
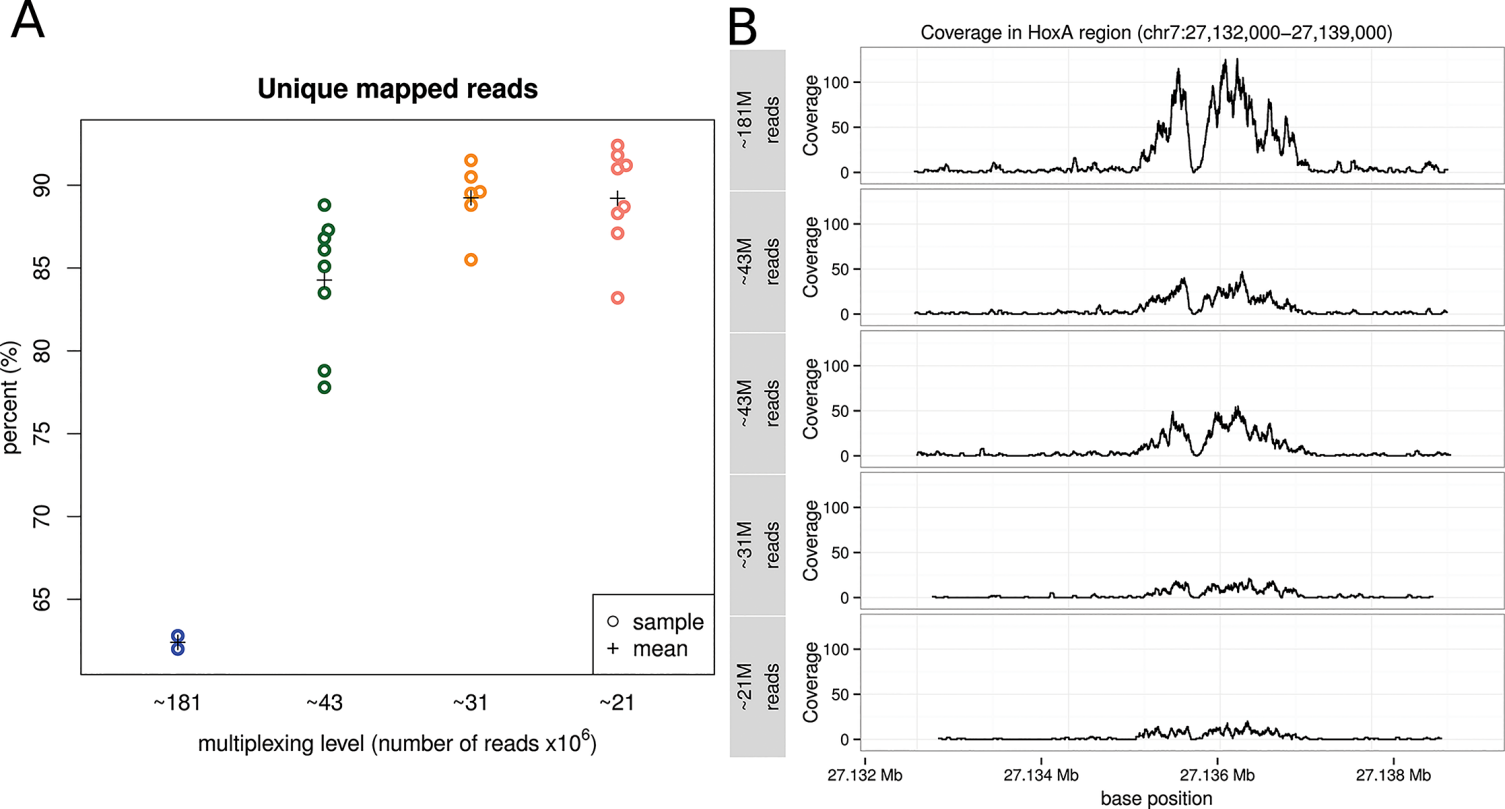
Mapped reads summary. A) Unique mapped reads are considered to be the fraction of mapped reads after duplicate reads are removed. Multiplexed libraries yield proportionally more unique mapped reads per lane. B) Genome view of sequence coverage along the HoxA region (chr7: 27,132,000–27,139,000) showing consistent coverage across all multiplex levels with decreasing coverage as multiplexing increases.

An average of 216 million single 50bp reads per lane for 6 lanes were generated with greater than 92% of the reads passing filter and greater than 96% of the bases having a quality score above Q30 (mean quality score Q37.8). As a first step, to determine whether there was sequencing consistency across the differently multiplexed libraries, we evaluated the number of reads generated by each library and the number of duplicated (identical) reads. Multiplexed libraries yielded an average of 26.4% more total reads and 45.1% less duplicated reads suggesting there is read saturation in libraries sequenced at 1-plex (Table 1). This contributes to an increase in the number of usable reads–the fraction of uniquely mapped reads–in the multiplexed samples (Fig 2A).

**Table 1:** Read counts and proportions.

Reads x10^6^ (multiplex factor)	Label	Total reads	% Pass filter	% of > = Q30 Bases (PF)	Mean quality score (PF)	Mapped reads	Clonal	Usable reads	Usable/Mapped (%)	Usable/Total reads (%)
~181 (1)	ChIP-2	180,919,574	94.21	97.14	38.2	141,334,233	52,600,090	88,734,143	62.8	49
	Input-2	186,914,799	90.72	96.07	37.76	142,235,243	54,102,709	88,132,534	62	47.2
~43 (4)	ChIP-6	65,994,330	90.88	95.57	37.55	50,506,894	7,509,321	42,997,573	85.1	65.2
	ChIP-12	44,766,472	91.57	95.7	37.6	35,070,086	4,863,613	30,206,473	86.1	67.5
	Input-4	82,954,090	90.66	95.75	37.61	60,722,599	13,471,137	47,251,462	77.8	57
	Input 5	58,158,842	91.54	95.95	37.69	44,471,553	5,855,499	38,616,054	86.8	66.4
~43 (4)	ChIP-4	78,851,924	92.98	96.34	37.86	60,667,164	9,997,778	50,669,386	83.5	64.3
	ChIP-5	53,074,973	93.54	96.52	37.94	41,493,467	5,275,151	36,218,316	87.3	68.2
	Input-6	46,404,486	93.16	96.66	38.01	35,457,222	3,961,293	31,495,929	88.8	67.9
	Input-12	43,262,826	93.5	96.72	38.04	33,989,221	7,191,470	26,797,751	78.8	61.9
~31 (6)	ChIP-2	31,186,192	93.07	96.31	37.84	24,279,056	2,518,570	21,760,486	89.6	69.8
	ChIP-4	48,602,375	92.8	96.3	37.84	37,383,166	4,198,375	33,184,791	88.8	68.3
	ChIP-5	37,480,885	93.13	96.47	37.91	29,241,485	2,786,055	26,455,430	90.5	70.6
	ChIP-7	44,486,053	92.8	96.32	37.85	34,242,835	4,966,694	29,276,141	85.5	65.8
	ChIP-12	31,695,051	93.15	96.45	37.91	24,989,189	2,628,874	22,360,315	89.5	70.5
	Input-6	33,223,876	92.96	96.62	37.98	25,361,883	2,144,548	23,217,335	91.5	69.9
~21 (8)	ChIP-1	27,745,182	92.91	96.31	37.84	21,283,791	3,568,944	17,714,847	83.2	63.8
	ChIP-2	23,401,070	93.32	96.28	37.83	18,249,468	1,494,474	16,754,994	91.8	71.6
	ChIP-3	24,674,925	92.57	95.95	37.68	18,778,438	2,416,530	16,361,908	87.1	66.3
	ChIP-4	36,312,228	92.95	96.26	37.83	27,965,673	2,472,080	25,493,593	91.2	70.2
	ChIP-5	28,530,578	93.22	96.42	37.9	22,268,041	1,692,055	20,575,986	92.4	72.1
	ChIP-6	35,095,024	92.88	96.29	37.84	27,104,039	2,432,794	24,671,245	91	70.3
	ChIP-7	32,766,925	92.9	96.27	37.83	25,245,427	2,846,520	22,398,907	88.7	68.4
	Input-12	21,184,781	93.4	96.65	38.01	16,638,484	1,949,311	14,689,173	88.3	69.3

The table displays the total number of reads sequenced, percent of reads that passed filter, percent of bases above quality score Q30, the mean quality score, number of mapped reads, number of reads considered clonal, number of usable reads, the proportion of mapped reads considered usable, and the proportion of total reads considered usable, grouped by multiplexing level.

A common and useful approach to assess the general quality of a ChIP-seq experiment is to visualize the mapped reads of some known sites at specific genomic location. We examined the coverage along the HoxA region (Chromosome 7: 27,132,000–27,139,000) and found that read coverage is consistent across all multiplexing levels with decreasing coverage as multiplexing is increased (Fig 2B).

### Impact of multiplexing on Peak Detection and Peak Characteristics

**Fig 3:**
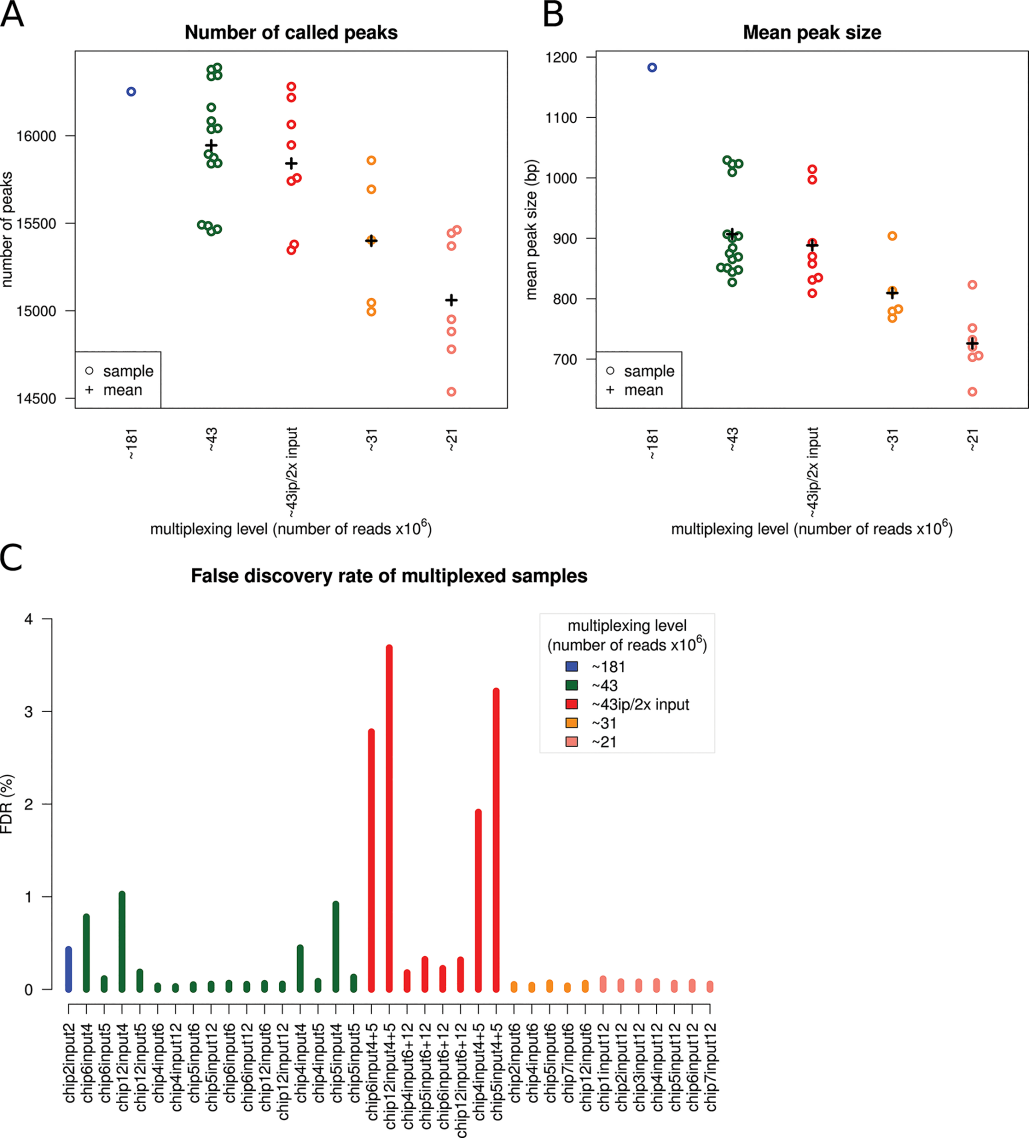
Peak detection and false discovery rate. As expected the number of peaks and their width are reduced as coverage is reduced. A) The mean number of peaks identified for each sample by multiplex level. B) The mean peak width of identified peaks for each sample by multiplex level. C) The false discovery rate (FDR) for each sample was computed by contrasting the input to the IP samples: ~181 million (M) reads (blue), ~43M reads (green), ~43M reads chip-2x input (red), ~31M reads (orange), ~21M reads (salmon). FDR for some experiments related to Input-4 are close to >0.45% suggesting these rates are an artifact of the library. Overall, experiments either 1-plex or multiplexed, with equal fractions of IP and input or double input, the FDR is < = 0.43%.

Sequencing depth for peak detection was within the range for sufficient ChIP signal strength (>20M mapped reads) defined in the guidelines by The ENCODE and modENCODE consortia [3]. Peaks were identified using default parameters of ChIPseeqer [4]. Using the peaks detected from the full IP (the reference set; 1-plex; ~181M reads) and input lanes, we compared the peaks detected from the multiplexed ChIPs (4-plex ~43M reads, 6-plex ~31M reads, and 8-plex ~21M reads). To determine the effect of reduced sequence coverage on peak discovery we counted the total number of peaks identified for each multiplexing level by the ChIPseeqer peak detection algorithm. For our initial approach, we averaged the number of peaks for each sample of each multiplexing level. As expected, reduced coverage as result of increasing the multiplexing factor results in fewer detected peaks. Compared to 1-plex, when using ~43M reads there were 1.9% fewer peaks, and when using ~21M reads there were 7.3% fewer peaks (Fig 3A), as well as reduction in the average peak width, 23.3% shorter width at ~43M reads and 38.6% shorter width at ~21M reads (Fig 3B). Since peak-calling algorithms can have very different models we repeated this analysis using MACS2 (an updated version of MACS; https://github.com/taoliu/MACS) [5]. When running MACS2 with parameter values estimated to be analogous to ChIPseeqer, MACS2 tended to call a greater number of peaks with shorter widths with the same trend of reduction in number or width as multiplexing increases (S1 and S2 Figs).

To assess the relative confidence in the sets of peaks called for each experiment we computed an empirical false discovery rate (FDR) by inverting the peak calling procedure such that ChIP peaks where used as background for calling significant peaks from input reads. In this scheme, any peaks that pass the statistical threshold for significance are considered false calls as they are enriched in the input samples over the IP samples. We applied this procedure using different ratios of input and ChIP libraries. FDR for some experiments (FDR > = 0.45%) appear to be related particularly to Input-4 suggesting these rates are an artifact of the library. Overall, empirical FDR results from 1-plex or more multiplexed libraries with equal fractions of input or double input, was < = 0.43% (Fig 3C).

**Fig 4:**
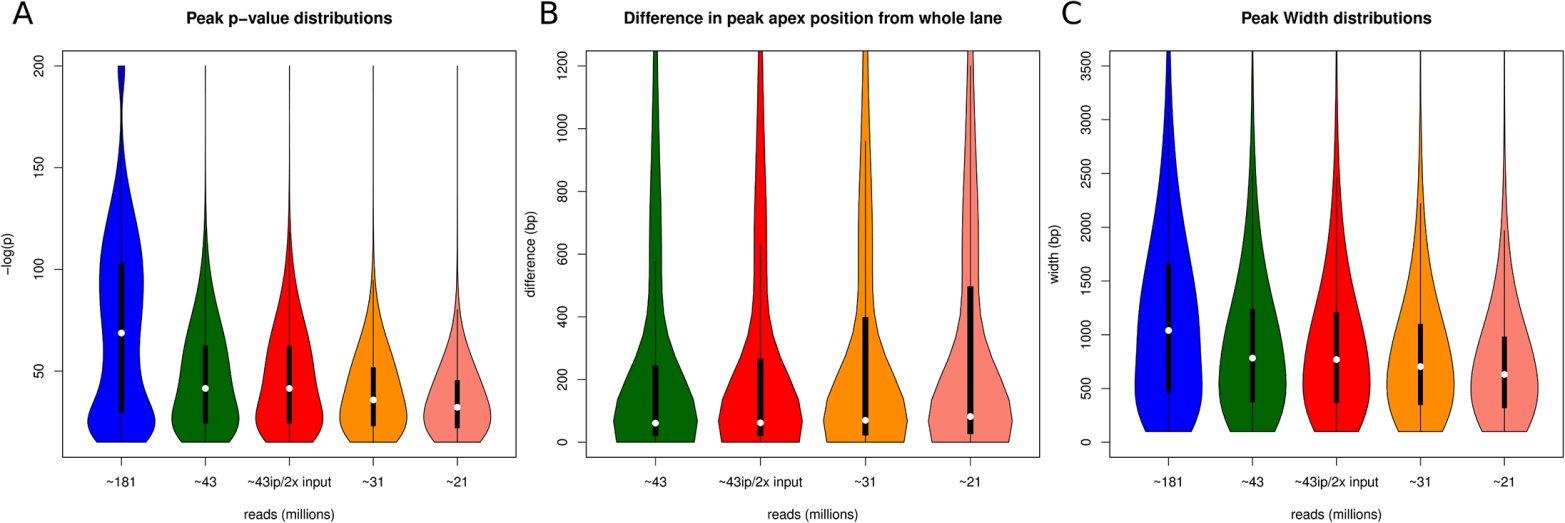
Peak characteristics. A) P-values for detected peaks shift towards reduced significance as multiplexing increases. B) The difference in peak apex position of peaks detected in multiplexed libraries to peak apex positions of peaks detected in the non-multiplexed library shows consistent difference across all multiplexed levels while increasing variability as multiplexing increases. C) Peak width distributions show a marginal reduction across multiplex levels.

Initially we compared the number of peaks and average peak widths to see if there were any critical differences. To understand in greater detail the variability of called peaks for each lane fraction, we examined the distributions of three peak characteristics: peak p-values, peak apex position, and peak widths. The p-values distributions show an overall shift towards reduced significance (higher p-values) as multiplexing increases (Fig 4A and S3 Fig) due to reduction in sequence coverage. While there is a significant overlap among peaks identified from multiplexed libraries with those identified from the non-multiplexed library we were interested to what extent the positions (genomic coordinates) of the overlapping peaks have shifted. Comparing the peak apex positions of peaks identified from 1-plex, most peaks, 88.1–85.1% (differences increase as multiplexing increases), were within the mean median peak size (751bp), suggesting little difference in peak positions (Fig 4B and S4 Fig). Libraries sequenced at higher multiplexing show more variability in peak apex position, possibly due to less data supporting a peak for detection. Finally, for each multiplex level we also examined the peak width distribution of identified peaks and found marginal reduction in peak width across multiplexed libraries (Fig 4C and S5 Fig). Taken together these results indicate that while the number of significant peaks identified is reduced due to multiplexing there is very little effect on the uniformity and positional coverage of H3K4me3.

### Impact of multiplexing on variability of peak detection and gene annotation

To evaluate the variability in peak detection we compared the peaks detected in each of the multiplexed samples to those identified by the 1-plex sample as well as comparing the peaks detected for each sample to each other sample for the same multiplex factor. The percent overlap is the number of overlapping peaks divided by the total number of peaks in the sample dataset. For example, given sample datasets X and Y, the percent overlap of X is the defined simply as |x ∩ y| / |x|–number of overlapping peaks between X and Y divided by the total peaks in X where overlap is defined as minimal 1 base overlap.

**Fig 5:**
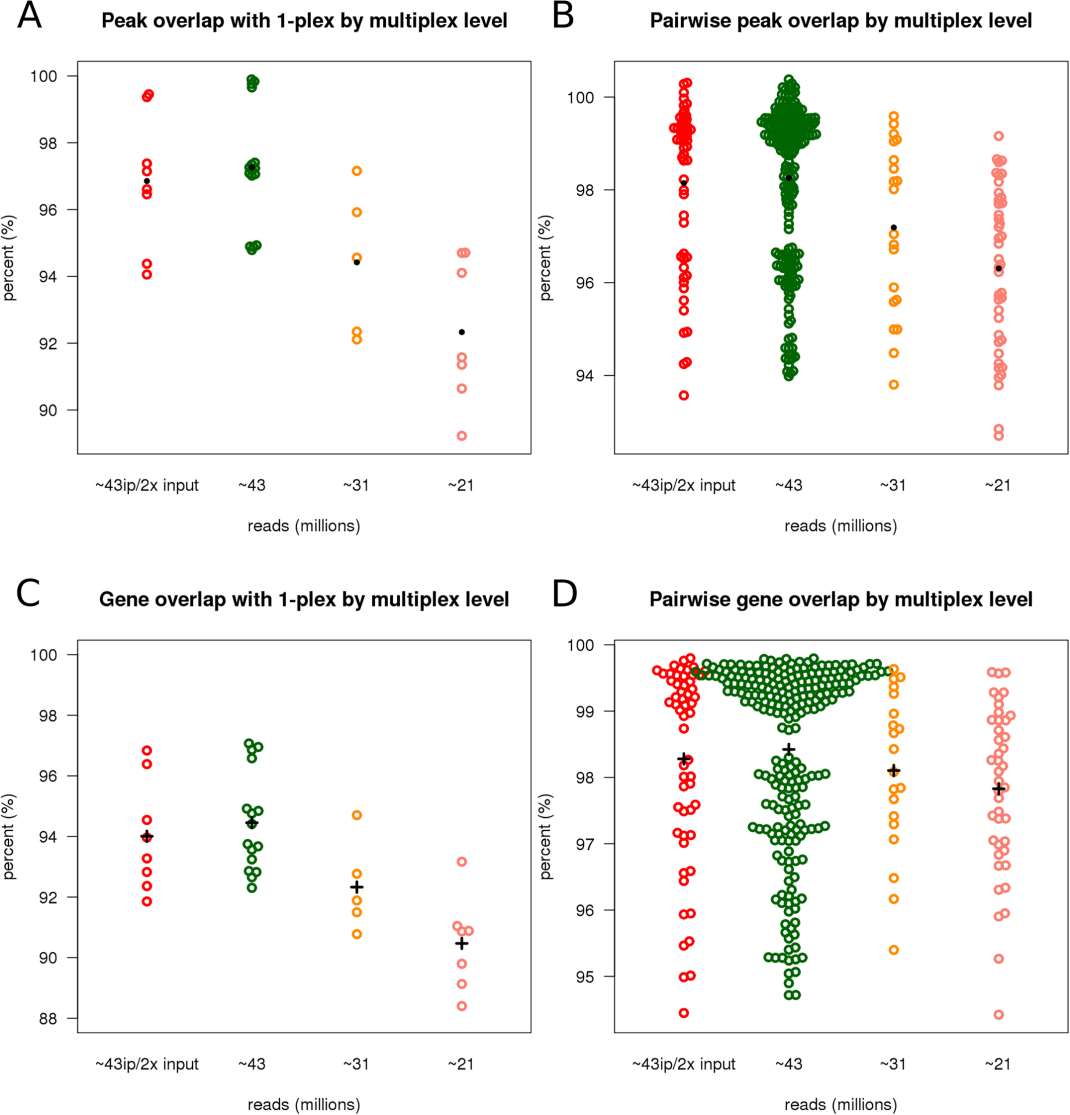
Overlap of detected peaks and gene annotation overlap across multiplex levels. A) The percent overlap of peaks for each sample to 1-plex across multiplex levels. As multiplexing increases there is a shift towards decreasing overlap. However, the overlap of peaks called from multiplexed samples with peaks identified in the non-multiplexed sample is generally above 92%. B) The percent overlap of each sample pairwise across multiplexed libraries. Overlap among peaks called from samples with similar coverage is generally above 94%. There is a similar trend, as in A, that as multiplexing increases overlaps start to decrease. C) The overlap of gene annotations of peaks called from each multiplexed sample compared to peaks from the non-multiplexed sample. Generally, as multiplexing increases more than 90% of the genes annotated in 1-plex are present in the gene annotations of multiplexed libraries. D) The percent of overlap of gene annotations pairwise for each sample.

Fig 5A shows the pairwise comparison of overlapping peaks of each sample to the 1-plex sample per multiplexing level. The mean overlap per multiplexing level is generally above 92% for all multiplexing levels and as expected the percent of overlapping peaks decreases as multiplexing increases. A similar trend of increased multiplexing and slight decrease in percent overlap is seen in pairwise comparisons among all samples, but the mean overlap by multiplexing level is above 96% (Fig 5B).

### Overlap of genes annotated from detected peaks

We performed gene-level annotation of the peaks detected by ChIPseeqer with ChIPseeqerAnnotate [4] on samples of all multiplexing levels and compared the pairwise overlap of annotated genes. Using the 1-plex sample as the reference for the number of genes annotated, we computed what percent of genes were annotated in each multiplexing level. In general, more than 90% of the genes annotated at 1-plex were also present in each multiplexing level (Fig 5C). As in other measures, the multiplexed gene sets overlap with full lane data gradually decreases from 95% to 90% as multiplexing increases. Intra-lane variability also indicates consistent gene set identification among replicated datasets of equal multiplexing degree (Fig 5D). We observe similar results when using MACS2 for peak detection (S6 and S7 Figs).

**Fig 6:**
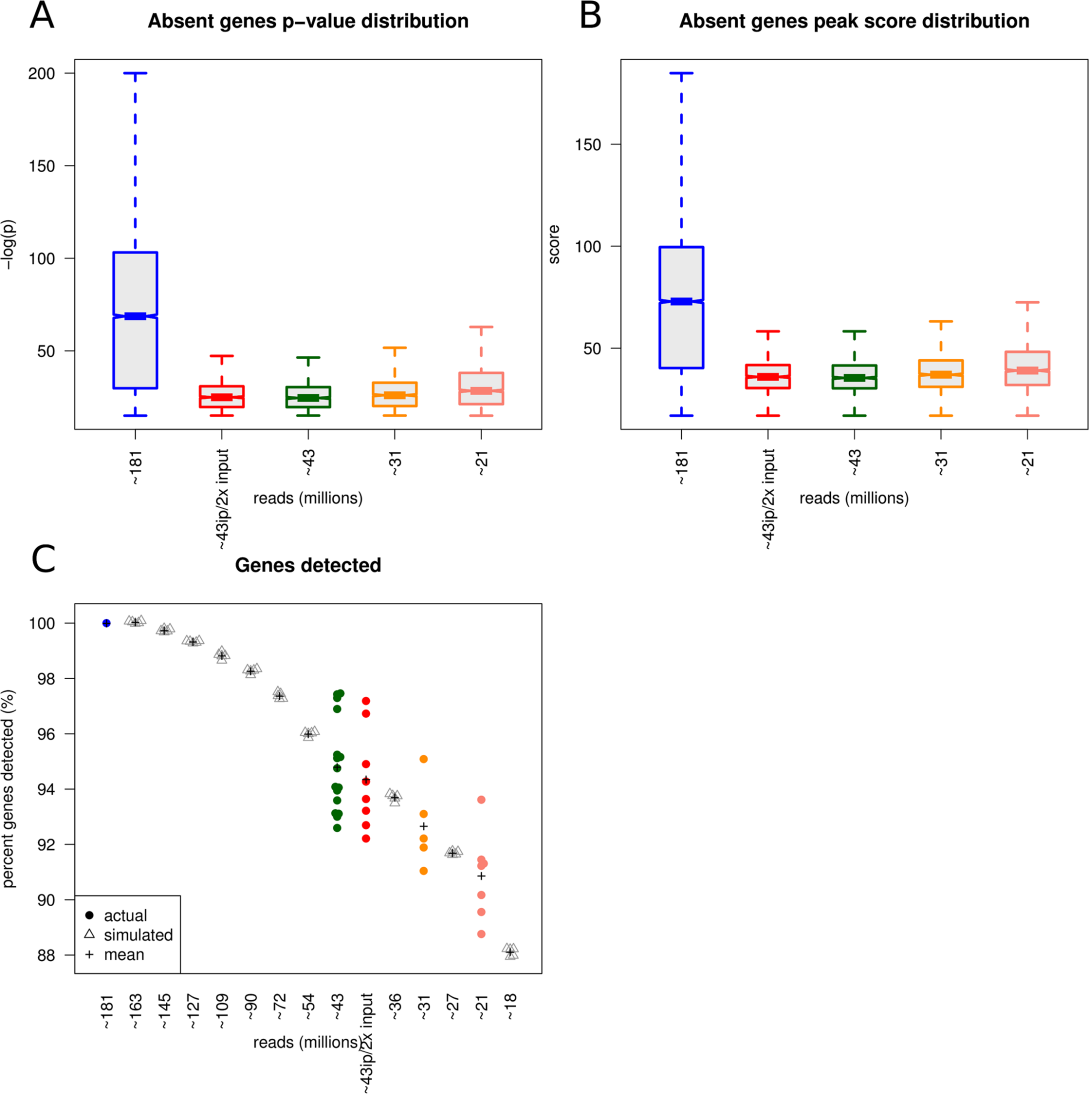
Characteristics of peaks lost due to reduced coverage and gene anntations of peaks from simulated reduced coverage. A) P-value distributions of peaks from gene annotations absent from multiplexed libraries showing the genes lost due to multiplexing having less significant peak p-values and appearing in the lower quartile of the 1-plex distribution. B) Peak width distributions of genes absent from multiplexed libraries showing that genes lost due to multiplexing having shorter peak widths and appearing in the lower quartile of the 1-plex distribution. C) Multiplexing was simulated by downsampling the non-multiplexed sample data in 10% increments from 90% to 10% in 5 replicates. There is strong agreement among the experimental and simulated data showing a gradual decrease in the number of gene annotated peaks as multiplexing increases and multiplexing to ~18M reads (currently 10% of a lane) can recover 88% of gene annotated peaks.

Next, we sought to characterize the impact of multiplexing on gene annotation and more specifically delineate the ChIP-seq characteristics for those annotated genes that are lost due to multiplexing. Explicitly, we identified the peaks missing from each multiplexing level relative to the 1-plex sample and examined the characteristics of those peaks (peak’s p-values and detection scores) in the 1-plex data. Generally, peaks that are dropped from detection by multiplexing are in the lower quartiles of the p-value and score distributions (Fig 6A and 6B), suggesting these peaks were weakly supported by the data and likely contained high fraction of fortuitous binding events.

### Lane fraction simulation

At the experimental level, in our model system, data obtained from a library sequenced at ~21M reads is comparable to the same library sequenced at ~181M reads. We sought to observe this *in silico* and ran a simulation to examine the effect of multiplexing ChIP-seq samples on gene annotation and compiled it with our experimental data. Multiplexing was simulated by downsampling the 1-plex data in 10% increments from 90% to 10% in 5 replicates. Then we performed the same peak detection, annotation, and percent recovery as we performed for the experimental data. We see a strong agreement among the experimental and simulated data (Fig 6C). The experimental 4-plex (~43M reads) samples fell between the 20% and 30% simulated multiplex factors, 6-plex (~31M reads) samples fell between 15% and 20% simulated multiplex factors, and the 8-plex (~21M reads) samples fell between the 10% and 15% simulated multiplex factors. Importantly, 88% of the annotated genes identified from ~181M reads (1-plex) were identified when multiplexing to ~18M reads (10% of a lane). In order to determine if these trends are specific to H3K4me3 and our cell lines, we collected publically available data for two other histone marks and a transcription factor. We compared our results for H3K4me3 in OCI-LY7 cells to the gene annotations of peaks called by ChIPseeqer on collected data for the same histone mark (H3K4me3) and two other histone marks (H3K27ac and H3K4me1) from MOLM1 cells [6], and to gene annotations of peaks on collected data for a transcription factor BCL6 from primary CXCR5hi CD4 T cells [7]. Data for ~43, ~31, and ~21 million reads were simulated by downsampling, as the initial data from these samples had sufficient reads yet were not originally sequenced with varying levels of coverage. Compared to OCI-LY7 H3K4me3, MOLM1 H3K4me3 and H3K27ac displayed similar results in recovery of gene annotations, 87.9% and 81.5% at ~21M reads, respectively, whereas MOLM1 H3K4me1 recovered just 14% of gene annotations at ~21M reads. Transcription factor BCL6 also showed a large decrease in gene annotations at ~21M reads with 24.7% for replicate 1 and 19.3% for replicate 2. Overall, the trend of the gene annotation analysis is consistent with our H3K4me3 results showing reduced recovery of peak's gene annotations as multiplexing increases, however, the impact on gene annotations depends on the factor analyzed (S8 and S9 Figs).

## Methods

### Chromatin Immunoprecipitation

Diffuse large B-cell lymphoma (DLBCL) cell line OCI-Ly7 (ACC688 Deutsche Sammlung von Mikroorganismen und Zellkulturen Braunschweig, Germany) was grown in medium containing 90% Iscove's, 10% fetal calf serum and supplemented with penicillin G and streptomycin. Immunoprecipitation using anti-histone H3 trimethyl K4 (ab8580, Abcam, Cambridge, MA) was performed in crosslinked sonicated extracts. Cells were grown at 1–1.5 million per ml and protein and DNA were cross-linked for a 10 minute treatment with 1% Formaldehyde (Thermo Scientific, Rockford, IL) and harvested. 100x10^6^ cells were lysed by flash freezing in liquid N_2_ and stored at -80°C. Chromatin shearing was performed using a Covaris S220 sonicator (Covaris, Woburn, MA) using the following conditions: 1 ml tubes with approximately 30 million cells in buffer containing 0.1% SDS (Covaris Buffer D3) were sonicated using peak intensity power of 140, duty factor of 5.0 and 200 cycles per burst, for 18 minutes. Extent of shearing was monitored with a 1% agarose gel and confirmed by separation on an 2100 High sensitivity Bioanalyzer chip (Agilent Technologies, Santa Clara, CA) at the completion of the immunoprecipitation. Immunoprecipitation was carried out at 4°C with overnight incubation using 97.5x10^6^ cell equivalents of chromatin and 15ug of antibody. Immunoprecipitates were captured with Protein A Dynabeads (Novex Life Technologies, Grand Island, NY), washed, and resuspended in buffer containing 100ug of RNAse A and 20ug of PCR grade Proteinase K (Qiagen, Germantown, Maryland). Crosslinks were reversed by incubation at 65°C in the presence of 1% SDS and 0.3M NaCl. Chromatin was then subjected to phenol:chloroform extraction followed by purification using a PCR purification kit (Qiagen, Germantown, Maryland). Quantitative real time PCR (qRT-PCR) on positive housekeeping genes was performed on both input and eluted chromatin, to validate the ChIP efficacy.

### Library preparation and Sequencing

Libraries compatible with Illumina TruSeq adapter sequencing (Illumina Inc, San Diego, CA) were made as follows: 10 ng of either immunoprecipitated or input DNA were end-repaired using 3 units of T4 DNA polymerase, 1 unit of Klenow DNA polymerase and 10 units of T4 DNA polymerase with 30 minutes incubation at 20°C; A-tailing was performed with 5 units of Klenow fragment and 10mM dATP (New England Biolabs, Ipswich, MA) for 30 minutes at 37°C. 2 ul of 1uM of annealed Illumina TruSeq adapters (Integrated DNA Technologies, Coralville, IA) were used in an overnight ligation at 4°C with 2000 units of T4 DNA Ligase. To account for the migration pattern of Y-forked Illumina adaptors, ligated products from 250–350bp were size selected in a 1% agarose gel. After purification, the PCR reaction was carried out with 300uM dNTP, 200uM of primers and 1 unit of Phusion High-Fidelity DNA Polymerase (Thermo Scientific, Rockford, IL). Initial denaturation of 94°Cx5 min, was followed by18 cycles of 94°Cx20secs, 60°Cx30secs, 72°Cx30secs, with a final extension/elongation step of 72°Cx5min. PCR product was cleaned by the use of SPRI beads as per manufacturer’s recommendation (Beckman Coulter, Indianapolis, IN). Final product was resuspended in 20 ul of TrisEDTA. Final yields were quantified in a Qubit 2.0 Fluorometer (Life Technologies, Grand Island, NY) and quality of the library was assessed on a DNA1000 Bioanalyzer chip (Agilent Technologies, Santa Clara, CA). Libraries were normalized to 2nM and loaded on an Illumina HiSeq 2500 at 6pM, per manufacturer’s recommended protocol for 50bp single-read runs. Illumina’s CASAVA 1.8.2 software was used to perform image capture, base calling and demultiplexing.

### ChIP-seq Quality Control

Variations in the quality and reproducibility of a ChIP experiment can be caused by a number of factors during sample preparation. Among them are DNA-protein cross-linking conditions, size of the sheared chromatin, antibody specificity, and quality of the library generated for sequencing [3, 8, 9]. In the experiments performed for this study we applied the following quality control steps: i) Cells were grown at log phase before crosslinking and fresh, methanol-free formaldehyde was used, ii) Sheared chromatin was evaluated using an Agilent 2100 Bioanalyzer High Sensitivity chip. The amount of DNA present at the size range required for library prep (130bp-230bp) was > 10% of the total sheared sample in order to obtain libraries that result in accurate representation of the original ChIP material, iii) Antibody used as per ENCODE project guidelines [3], Anti-Histone H3 (trimethyl K4)–Abcam ChIP Grade(ab8580) characterized by Abcam. iv) Success of ChIP was verified by using quantitative real time PCR (qRT-PCR) on positive control sites from housekeeping genes. We obtained a ratio of >10 in ChIP over IP enrichment, v) Libraries were evaluated for a) yields of 200ng-300ng final material, b) quality of library by size range of 250bp-350bp excluding presence of adaptors or primer dimers, and c) qRT-PCR, as in step iv, for housekeeping genes, and vi) After sequencing, duplication rates were estimated using FastQC[10], rates obtained were less than 40% duplicated reads. Note that this measure depends on the abundance of the histone modification and can vary depending on genomic representation present in the ChIP.

### Computational Analysis

Illumina’s CASAVA 1.8.2 was used to generate fastq files from basecalls and Elandv2 aligner was used to align the sequenced reads to the human genome hg19 build using default alignment parameters.

Peak identification was performed using ChIPseeqer [4] with the read length set to 50bp and default values for all other parameters (for example, peak significance value threshold 10^−15^ and ChIP peaks need to be at least 2 fold higher than input peaks). Gene annotation was performed with ChIPseeqerAnnotate (a ChIPseeqer tool) and peaks were assigned to genes if their genomic locations are within 1kb of transcriptional start site, or transcriptional end site.

Additional custom analysis scripts were written in R (version 2.15.2[11]), including Bioconductor [12] packages: Genomic Ranges [13], beeswarm[14], Rsamtools[15], and ggbio[16], were used to perform the analysis.

## Discussion

We performed a systematic analysis of the dependency between sequence depths and the amount and characteristics of the detected peaks in ChIP-seq experiments with the goal of identifying the optimal multiplexing scheme that maximizes the number of samples with minimal loss of biological information. There was little variation in total numbers of reads and quality scores among sequencing replicates indicating overall consistency in sequencing quality (although biological and library preparation variation can be more substantial) and multiplexed libraries yielded more usable reads proportionally, possibly due to decreased amount of redundant reads.

There was little variation in number of peaks discovered for each lane fraction across the variously multiplexed libraries (mean peak number = 15690 +/- 3% (492)), low false discovery rate, <1%, and consistent peak characteristics (peak widths, p-values, and apex positions). A high percentage (>90%) of peaks from multiplexed libraries overlapped with the reference set of peaks (peaks identified from 1-plex library). Together, these results demonstrate high sensitivity and reproducibility for detecting peaks in these data.

Gene-level annotation of the peaks revealed >90% overlap of the annotated genes across each multiplex level. However, as multiplexing increased there was a slight decrease in gene annotation overlap from ~97% for ~43M reads to ~92% for ~21M reads. Examining the properties of the annotated peaks that were lost due to multiplexing showed that the majority of those had weak evidence for the H3K4me3 marker in the ~181M read dataset. Simulation analysis of lane fractions from 90%-10% was consistent with experimental data and at simulated ~18M reads, >88% of the genes may still be detected in the data.

Sequencing a single library per lane yields greater coverage and depth than ChIP-ChIP [5,17,18], but also exceeds the needs of most experiments. Multiplexing ChIP-seq experiments is economical alternative to maximize the information yield from ChIP-seq experiments with minimal loss of biological signal. Our results indicate that sequencing 7 IP samples and one input sample (8-plex) on a single HiSeq2500 lane (~21M reads per sample using V3 chemistry) can still yield >90% of the information gained from a non-multiplexed sample of ~181M reads. We emphasize that these experiments were performed with highly specific antibody in optimized conditions against a well-defined histone modification. We examined additional datasets from other histone markers and transcription factor ChIP-seq experiments and found comparable results to those reported here. However, results may vary significantly when ChIP is performed with less specific antibodies. Nonetheless, this study provides a useful guide for designing future experiments for optimal and economical use of ChIP-seq assays.

## Supporting Information

S1 Fig Peak detection by MACS2. The mean number of peaks identified for each sample by multiplex level as number of reads.
(PDF)

S2 Fig MACS2 peak size. The mean peak width of identified peaks for each sample by multiplex level as number of reads.
(PDF)

S3 Fig Empirical cumulative distribution function (CDF) of peak p-value distributions. (PDF)

S4 Fig Empirical cumulative distribution function (CDF) of difference in peak apex position from 1-plex. (PDF)

S5 Fig Empirical cumulative distribution function (CDF) of peak width distributions. (PDF)

S6 Fig Gene annotation recovery of peaks detected by ChIPseeqer and MACS2 on experimental data by multiplexing level as number of reads. MACS2 parameters were estimated to be most similar to the parameters used for ChIPseeqer. Gene annotations of peaks called with MACS2 show a trend consistent with ChIPseeqer, but fewer gene annotations were detected by MACS2.
(PDF)

S7 Fig Comparison of overlap of unique gene annotations on peaks called by ChIPseeqer and MACS2. Fewer unique gene annotations were detected by MACS2 but there was a high degree of overlap (mean percent overlap = 95.8% +/- 4.7%).
(PDF)

S8 Fig Gene annotation recovery for different histone marks by multiplexing level as number of reads. We compared our results for OCI-LY7 H3K4me3 to the gene annotations of peaks called by ChIPseeqer on collected data for the same histone mark (H3K4me3) and two other histone marks (H3K27ac and H3K4me1) from a different cell line, MOLM1 [6]. Data for ~43, ~31, and ~21 million reads was simulated since these experiments were performed at ~205M reads for H3K27ac/H3K4me3 and ~197M reads for H3K4me1. For all datasets, the overall trend is consistent with H3K4me1 showing reduced recovery of peak's gene annotations.
(PDF)

S9 Fig Gene annotation recovery for or a transcription factor by multiplexing level as number of reads. We compared our results to the gene annotations of peaks on collected data for a transcription factor, BCL6. Data for ~43, ~31, and ~21 million reads was simulated since these experiments were performed at ~105M reads for BCL6 rep 1 and ~89M reads for BCL6 rep 2 [7]. The overall trend for the BCL6 data is consistent with our H3K4me3 experimental data with both replicates of BCL6 showing reduced recovery of peak's gene annotations.
(PDF)

S1 Table Intra-lane and inter-lane ChIP/input combinations for peak calling and comparisons. (PDF)
