# Supplementary material for: Multiplexing of ChIP-Seq Samples in an Optimized Experimental Condition Has Minimal Impact on Peak Detection
Source: PLoS One. 2015 Jun 11;10(6):e0129350. doi: 10.1371/journal.pone.0129350 (PMC4466019; doi:10.1371/journal.pone.0129350)

**Figure S2. MACS2 peak size.** The mean peak width of identified peaks for each sample by multiplex level as number of reads.

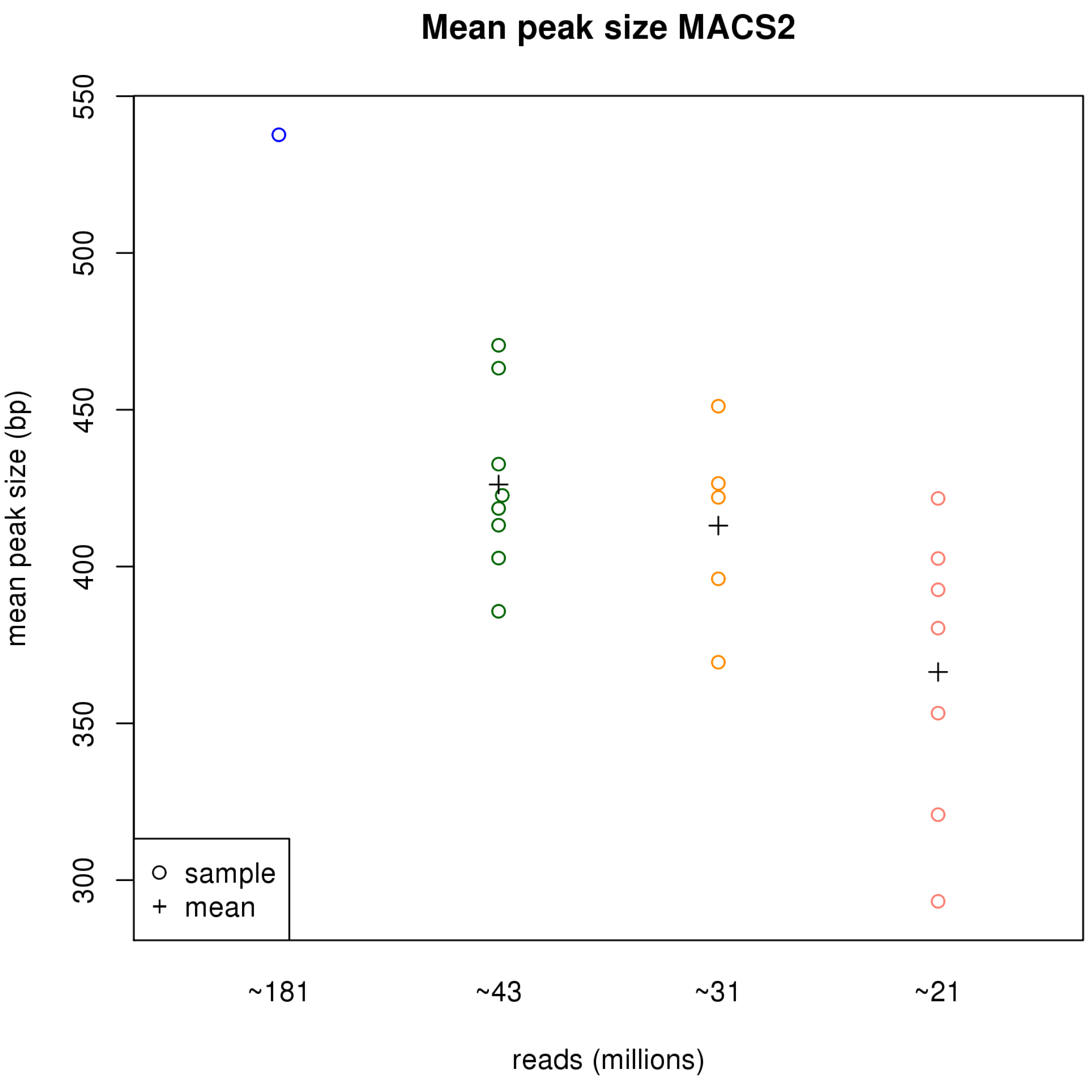

Supplement: S2 Fig — The mean peak width of identified peaks for each sample by multiplex level as number of reads. (PDF) [file pone.0129350.s002.pdf]
