# Supplementary figures and images for: Multiplexing of ChIP-Seq Samples in an Optimized Experimental Condition Has Minimal Impact on Peak Detection
Source: PLoS One. 2015 Jun 11;10(6):e0129350. doi: 10.1371/journal.pone.0129350 (PMC4466019; doi:10.1371/journal.pone.0129350)

**Figure S3. Empirical cumulative distribution function (CDF) of peak p-value distributions.**

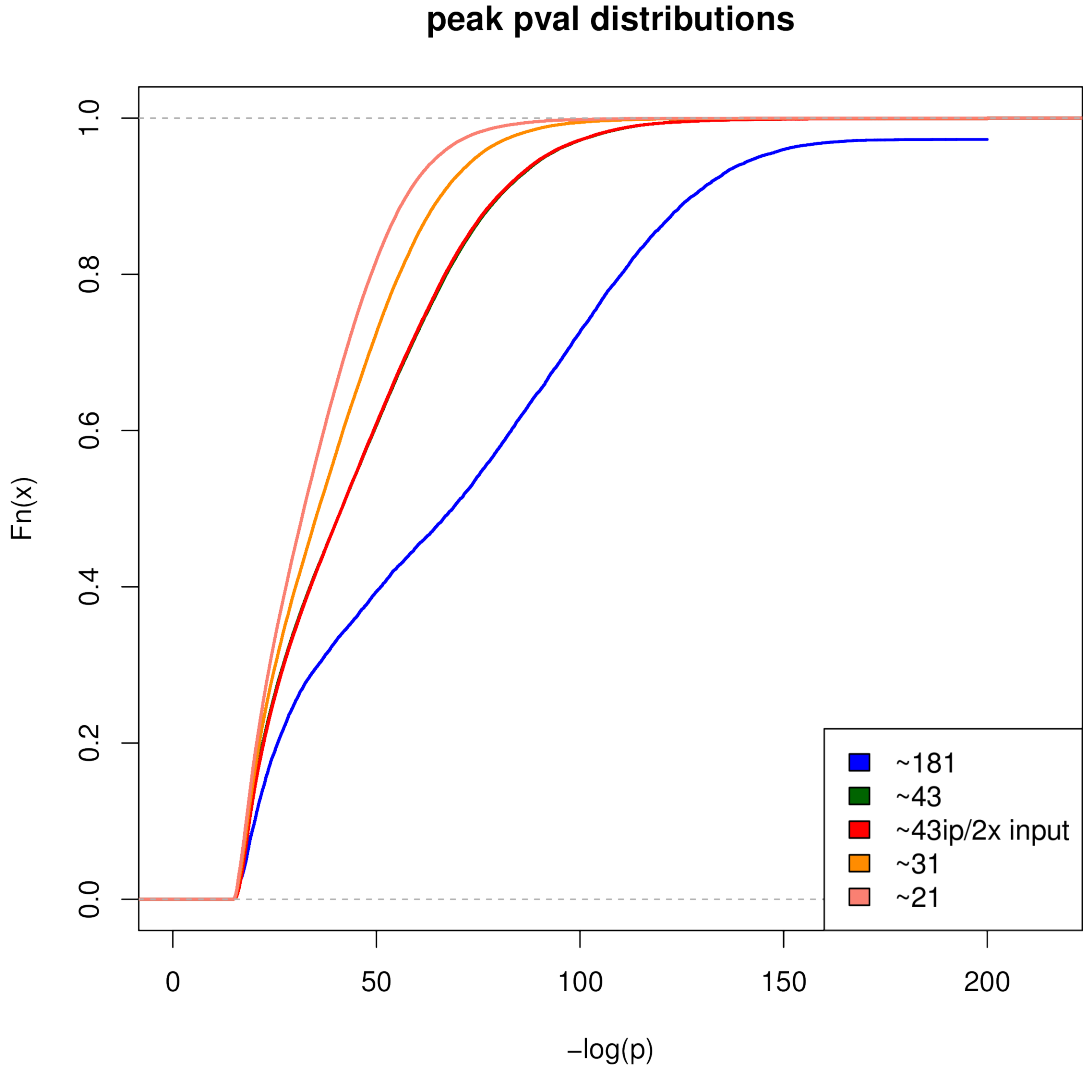

Supplement: S3 Fig — (PDF) [file pone.0129350.s003.pdf]

**Figure S5. Empirical cumulative distribution function (CDF) of peak width distributions.**

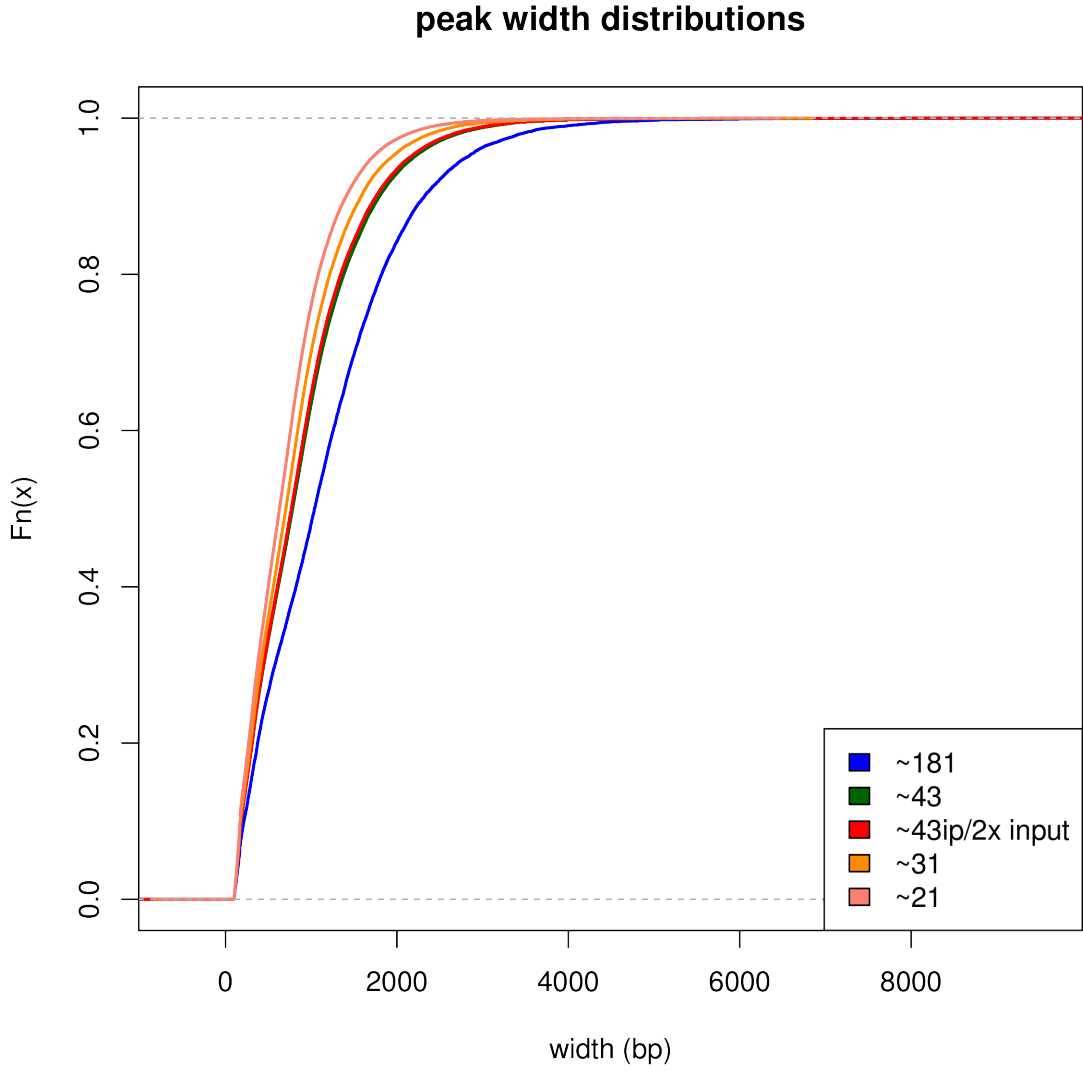

Supplement: S5 Fig — (PDF) [file pone.0129350.s005.pdf]
