# Supplementary material for: Multiplexing of ChIP-Seq Samples in an Optimized Experimental Condition Has Minimal Impact on Peak Detection
Source: PLoS One. 2015 Jun 11;10(6):e0129350. doi: 10.1371/journal.pone.0129350 (PMC4466019; doi:10.1371/journal.pone.0129350)

Figure S4. Empirical cumulative distribution function (CDF) of difference in peak apex position from 1-plex.

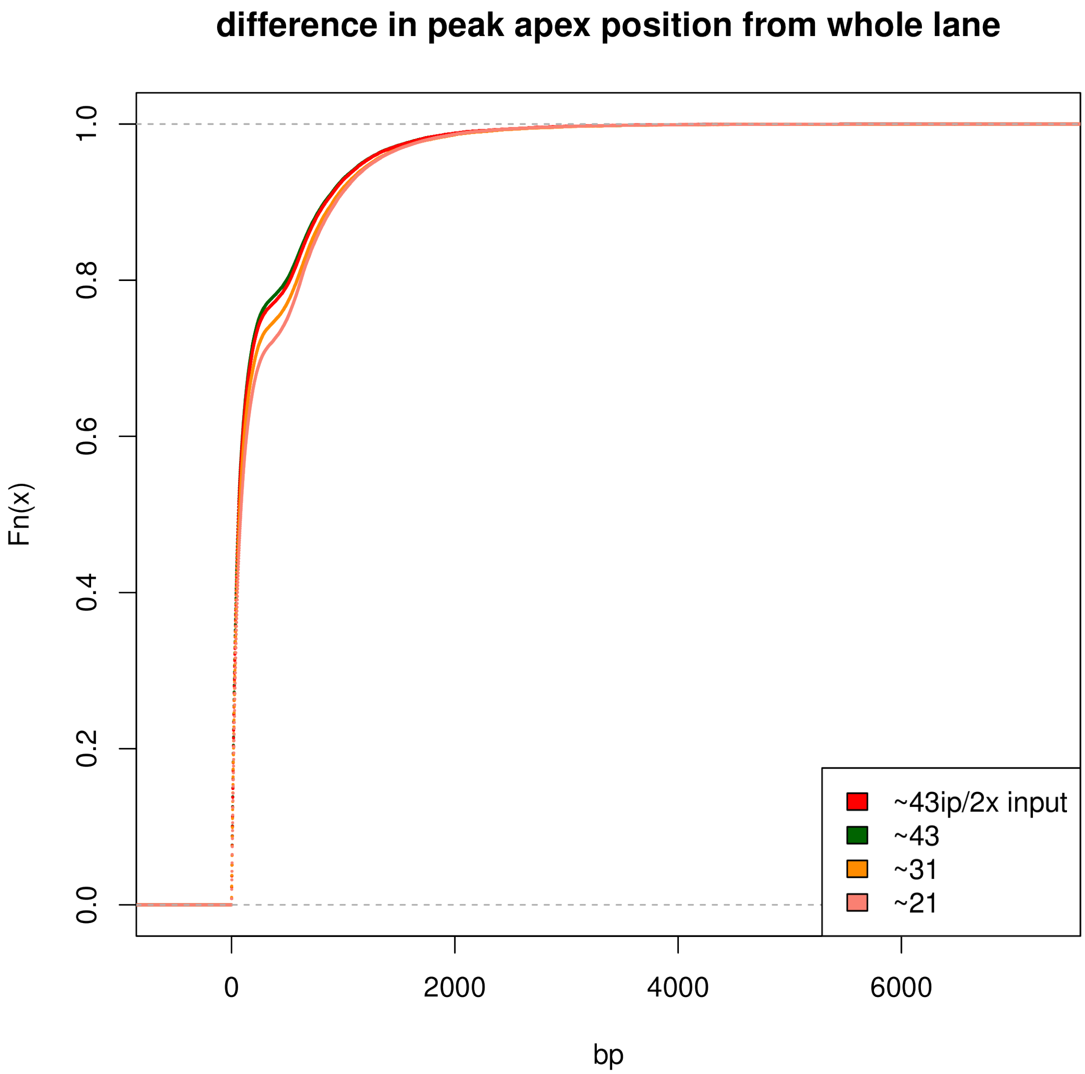

Supplement: S4 Fig — (PDF) [file pone.0129350.s004.pdf]
