# Supplementary material for: Multiplexing of ChIP-Seq Samples in an Optimized Experimental Condition Has Minimal Impact on Peak Detection
Source: PLoS One. 2015 Jun 11;10(6):e0129350. doi: 10.1371/journal.pone.0129350 (PMC4466019; doi:10.1371/journal.pone.0129350)

**Figure S6. Gene annotation recovery of peaks detected by ChIPseeqer and MACS2 on experimental data by multiplexing level as number of reads.** MACS2 parameters were estimated to be most similar to the parameters used for ChIPseeqer. Gene annotations of peaks called with MACS2 show a trend consistent with ChIPseeqer, but fewer gene annotations were detected by MACS2.

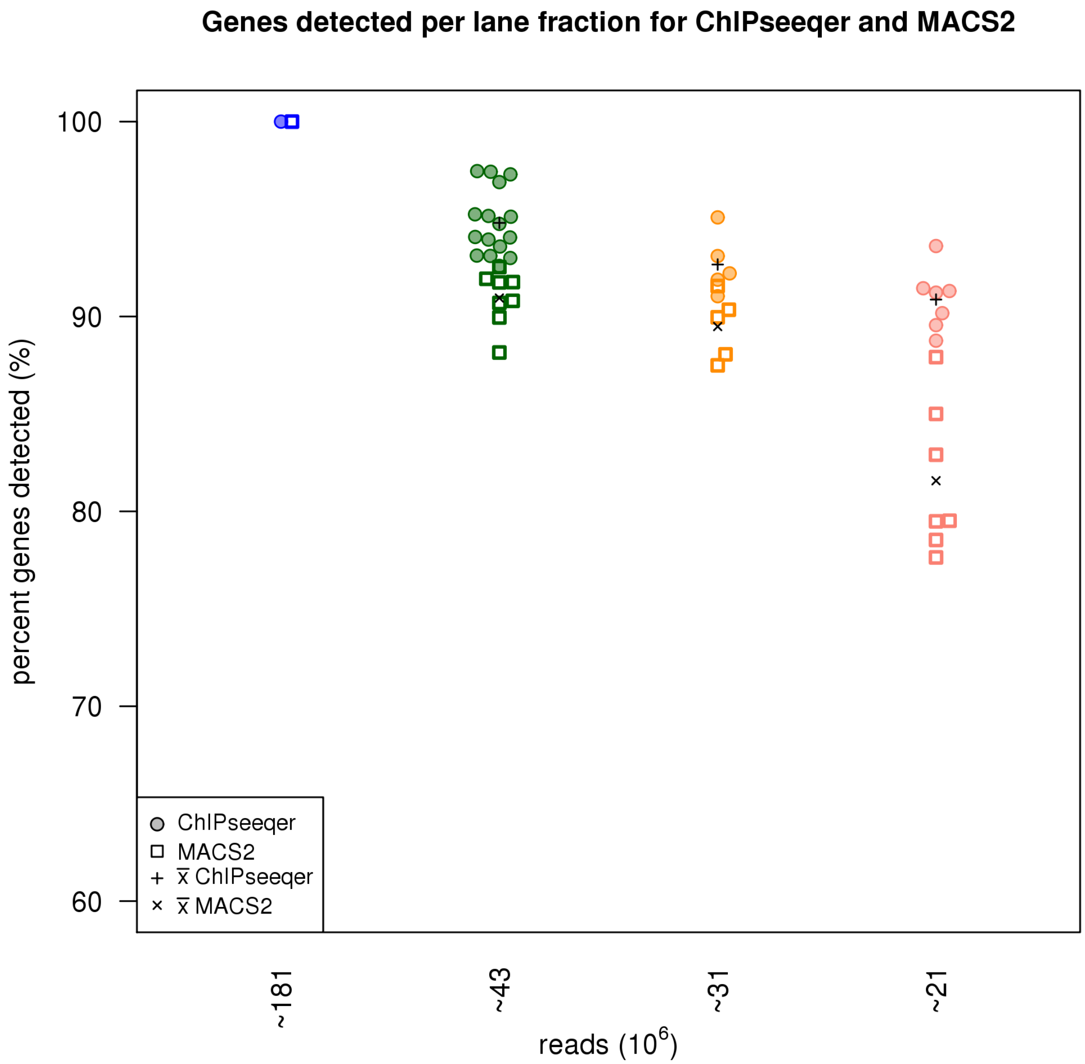

Supplement: S6 Fig — MACS2 parameters were estimated to be most similar to the parameters used for ChIPseeqer. Gene annotations of peaks called with MACS2 show a trend consistent with ChIPseeqer, but fewer gene annotations were detected by MACS2. (PDF) [file pone.0129350.s006.pdf]
