# Supplementary material for: Multiplexing of ChIP-Seq Samples in an Optimized Experimental Condition Has Minimal Impact on Peak Detection
Source: PLoS One. 2015 Jun 11;10(6):e0129350. doi: 10.1371/journal.pone.0129350 (PMC4466019; doi:10.1371/journal.pone.0129350)

Figure S7. Comparison of overlap of unique gene annotations on peaks called by ChIPseeqer and MACS2. Fewer unique gene annotations were detected by MACS2 but there was a high degree of overlap (mean percent overlap = 95.8% +/- 4.7%).

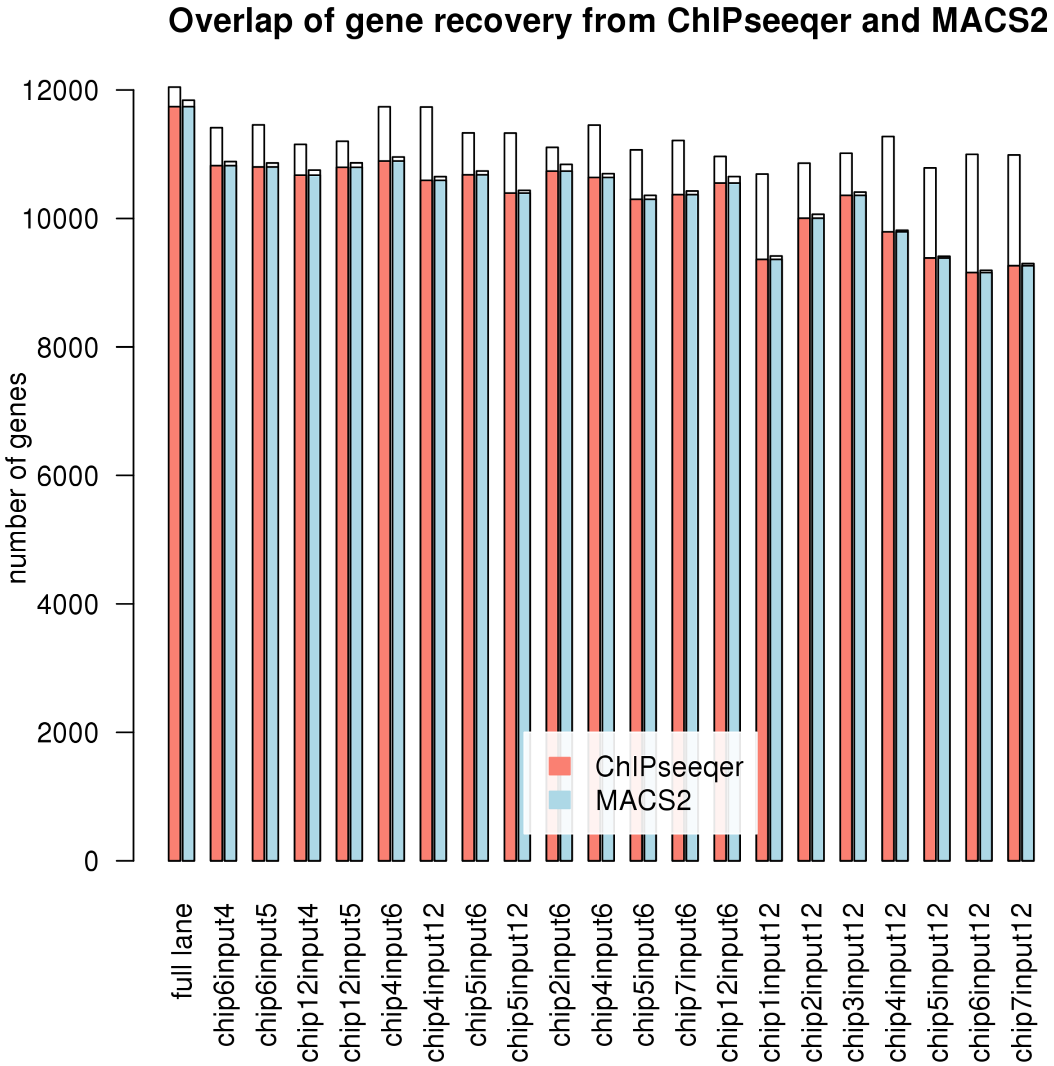

Supplement: S7 Fig — Fewer unique gene annotations were detected by MACS2 but there was a high degree of overlap (mean percent overlap = 95.8% +/- 4.7%). (PDF) [file pone.0129350.s007.pdf]
