# Supplementary material for: Multiplexing of ChIP-Seq Samples in an Optimized Experimental Condition Has Minimal Impact on Peak Detection
Source: PLoS One. 2015 Jun 11;10(6):e0129350. doi: 10.1371/journal.pone.0129350 (PMC4466019; doi:10.1371/journal.pone.0129350)

Figure S8. Gene annotation recovery for different histone marks by multiplexing level as number of reads. We compared our results for OCI-LY7 H3K4me3 to the gene annotations of peaks called by ChIPseeqer on collected data for the same histone mark (H3K4me3) and two other histone marks (H3K27ac and H3K4me1) from a different cell line, MOLM1 [6]. Data for ~43, ~31, and ~21 million reads was simulated since these experiments were performed at ~205M reads for H3K27ac/H3K4me3 and ~197M reads for H3K4me1. For all datasets, the overall trend is consistent with H3K4me1 showing reduced recovery of peak's gene annotations.

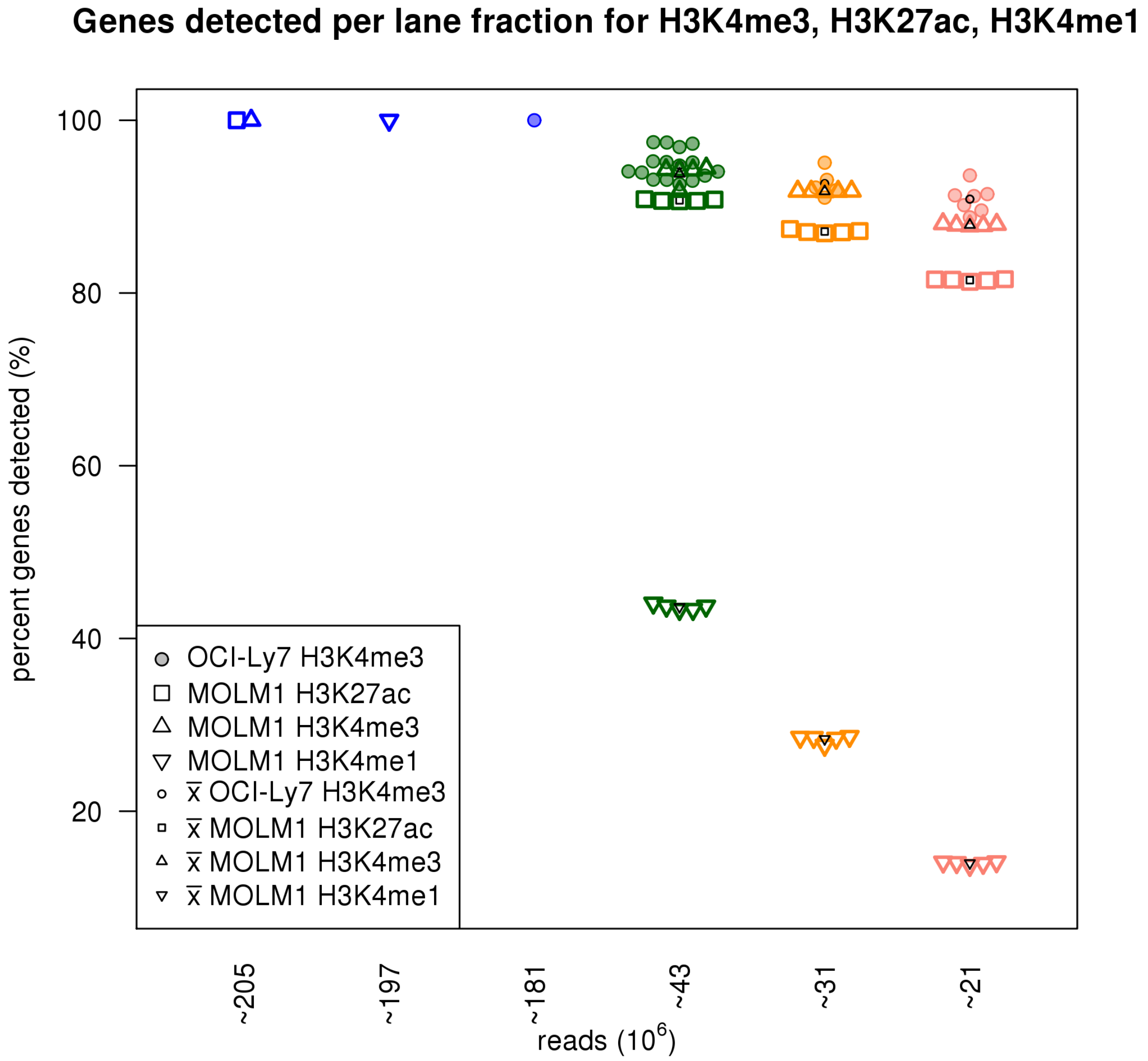

Supplement: S8 Fig — We compared our results for OCI-LY7 H3K4me3 to the gene annotations of peaks called by ChIPseeqer on collected data for the same histone mark (H3K4me3) and two other histone marks (H3K27ac and H3K4me1) from a different cell line, MOLM1 [6]. Data for ~43, ~31, and ~21 million reads was simulated since these experiments were performed at ~205M reads for H3K27ac/H3K4me3 and ~197M reads for H3K4me1. For all datasets, the overall trend is consistent with H3K4me1 showing reduced recovery of peak's gene annotations. (PDF) [file pone.0129350.s008.pdf]
