# Supplementary material for: Multiplexing of ChIP-Seq Samples in an Optimized Experimental Condition Has Minimal Impact on Peak Detection
Source: PLoS One. 2015 Jun 11;10(6):e0129350. doi: 10.1371/journal.pone.0129350 (PMC4466019; doi:10.1371/journal.pone.0129350)

Figure S9. Gene annotation recovery for or a transcription factor by multiplexing level as number of reads. We compared our results to the gene annotations of peaks on collected data for a transcription factor, BCL6. Data for ~43, ~31, and ~21 million reads was simulated since these experiments were performed at ~105M reads for BCL6 rep 1 and ~89M reads for BCL6 rep 2 [7]. The overall trend for the BCL6 data is consistent with our H3K4me3 experimental data with both replicates of BCL6 showing reduced recovery of peak's gene annotations.

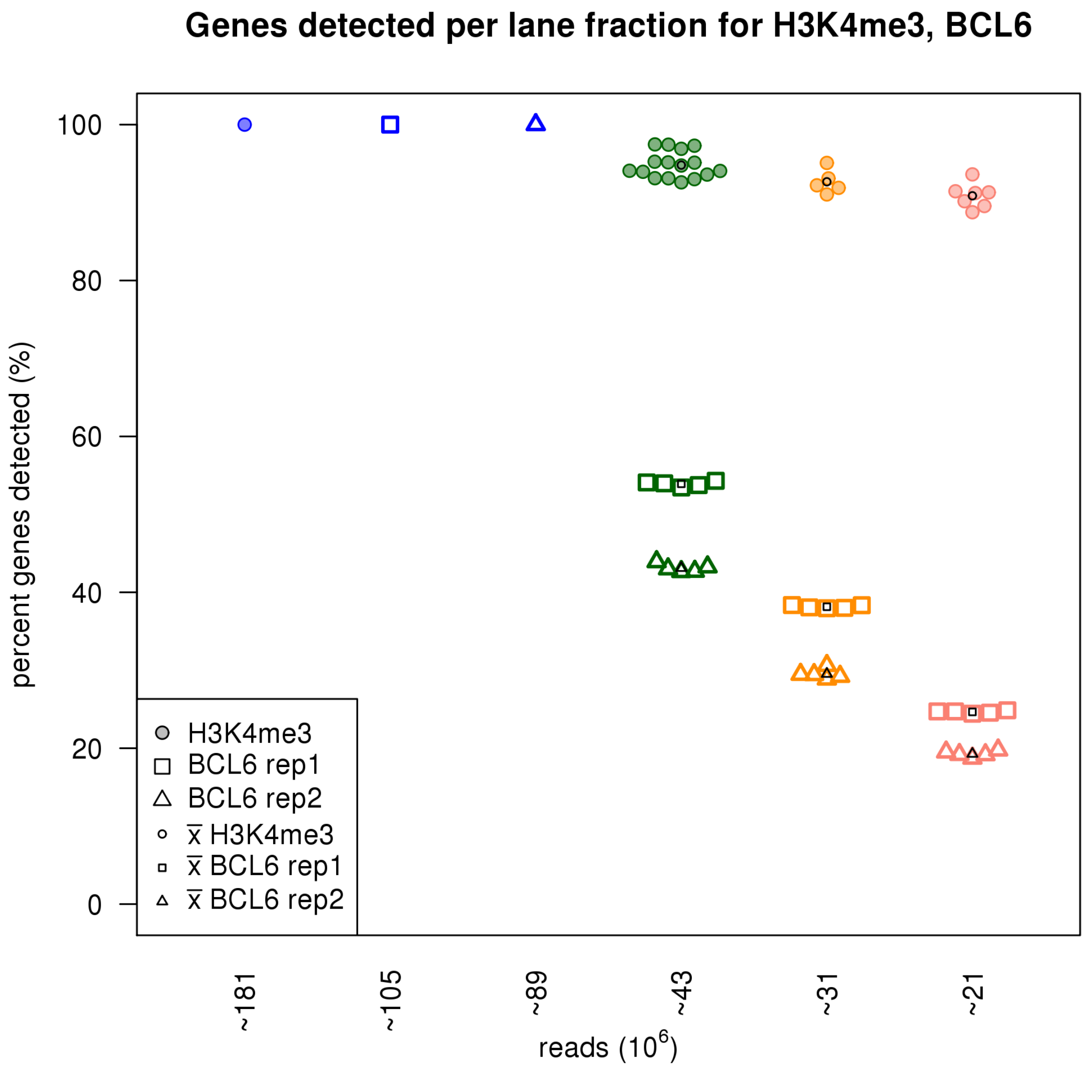

Supplement: S9 Fig — We compared our results to the gene annotations of peaks on collected data for a transcription factor, BCL6. Data for ~43, ~31, and ~21 million reads was simulated since these experiments were performed at ~105M reads for BCL6 rep 1 and ~89M reads for BCL6 rep 2 [7]. The overall trend for the BCL6 data is consistent with our H3K4me3 experimental data with both replicates of BCL6 showing reduced recovery of peak's gene annotations. (PDF) [file pone.0129350.s009.pdf]
