# Supplementary material for: Multiplexing of ChIP-Seq Samples in an Optimized Experimental Condition Has Minimal Impact on Peak Detection
Source: PLoS One. 2015 Jun 11;10(6):e0129350. doi: 10.1371/journal.pone.0129350 (PMC4466019; doi:10.1371/journal.pone.0129350)
Supplement: S1 Table — (PDF) [file pone.0129350.s010.pdf]

Table S1. Intra-lane and inter-lane ChIP/input combinations for peak calling and comparisons.

	Number of reads x10^6	ChIP/Input matching
Intra-lane	~181	chip-2::input-2
	~43	chip-6::input-4, chip-6::input-5, chip-12::input-4, chip-12::input 5, chip-4::input-6, chip-4::input-12, chip-5::input-6, chip-5::input-12
	~43ip/2x input	chip-4::input-6+input-12, chip-5::input-6+input-12, chip-6::input-4+input-5, chip-12::input-4+input-5
	~31	chip-2::input-6, chip-4::input-6, chip-5::input-6, chip-7::input-6, chip-12::input-6
	~21	chip-1::input-12, chip-2::input-12, chip-3::input-12, chip-4::input-12, chip-5::input-12, chip-6::input-12, chip-7::input-12
Inter-lane	~43	chip-6::input-6, chip-6::input-12, chip-12::input-6, chip-12::input 12, chip-4::input-4, chip-4::input-5, chip-5::input-4, chip-5::input-5
	~43ip/2x input	chip-4::input-4+input-5, chip-5::input-4+input-5, chip-6::input-6+input-12, chip-12::input-6+input-12
